# Euclidean Distances as measures of speaker similarity including identical twin pairs: A forensic investigation using source and filter voice characteristics

**DOI:** 10.1016/j.forsciint.2016.11.020

**Published:** 2017-01

**Authors:** Eugenia San Segundo, Athanasios Tsanas, Pedro Gómez-Vilda

**Affiliations:** a Department of Language and Linguistic Science, University of York, Heslington, York, YO10 5DD, UK; b Institute of Biomedical Engineering, Department of Engineering Science, University of Oxford, Oxford, UK; c Wolfson Centre for Mathematical Biology, Mathematical Institute, University of Oxford, Oxford, UK; d Sleep and Circadian Neuroscience Institute, Nuffield Department of Medicine, University of Oxford, UK; e NeuVox Lab, Center for Biomedical Technology, Universidad Politécnica de Madrid, Madrid, Spain

**Keywords:** Forensic phonetics, Acoustic analysis, Perceptual assessment, Voice quality, Twins, Pause fillers

## Abstract

There is a growing consensus that hybrid approaches are necessary for successful speaker characterization in Forensic Speaker Comparison (FSC); hence this study explores the forensic potential of voice features combining *source* and *filter* characteristics. The former relate to the action of the vocal folds while the latter reflect the geometry of the speaker’s vocal tract. This set of features have been extracted from pause fillers, which are long enough for robust feature estimation while spontaneous enough to be extracted from voice samples in real forensic casework. Speaker similarity was measured using standardized Euclidean Distances (ED) between pairs of speakers: 54 different-speaker (DS) comparisons, 54 same-speaker (SS) comparisons and 12 comparisons between monozygotic twins (MZ). Results revealed that the differences between DS and SS comparisons were significant in both high quality and telephone-filtered recordings, with no false rejections and limited false acceptances; this finding suggests that this set of voice features is highly speaker-dependent and therefore forensically useful. Mean ED for MZ pairs lies between the average ED for SS comparisons and DS comparisons, as expected according to the literature on twin voices. Specific cases of MZ speakers with very high ED (i.e. strong dissimilarity) are discussed in the context of sociophonetic and twin studies. A preliminary simplification of the Vocal Profile Analysis (VPA) Scheme is proposed, which enables the quantification of voice quality features in the perceptual assessment of speaker similarity, and allows for the calculation of perceptual–acoustic correlations. The adequacy of z-score normalization for this study is also discussed, as well as the relevance of heat maps for detecting the so-called *phantoms* in recent approaches to the biometric menagerie.

## Highlights

• Assessment of speaker similarity combining *source* and *filter* voice characteristics.
• Feature selection method to determine the most parsimonious feature subset.
• Testing with very similar-sounding speakers, i.e. monozygotic twins (MZ).
• Testing using high quality and telephone-filtered recordings.
• Significant differences between same-speaker and different-speaker comparisons.

## 1 Introduction

The human vocal apparatus is a sophisticated system relying on the accurate synchronization of multiple organic structures (e.g. lungs, vocal folds, vocal tract) in order to produce speech. Researchers from diverse disciplines have approached this field from very different angles, and continue contributing to the understanding of this immensely complicated process. Traditionally, the structures involved in speech production have been separated into the systemic view of the source-filter model due to Gunnar Fant [1], where the laryngeal structures are credited for the production of phonation, and the supralaryngeal structures are credited for modifying phonation spectral contents dynamically. Although early works relied on the study of phonated speech as a whole, over the last years there is a growing consensus that hybrid approaches that take into account the source-filter distinction are needed for achieving more reliable techniques in Forensic Speaker Comparison [2]; hence this study undertakes the analysis of a set of voice features combining *source* and *filter* characteristics of the human voice.

State-of-the-art research on twins’ voices [3], [4] suggests that distinguishing this type of speakers poses a major challenge in speaker recognition because they are very similar. Extreme physical similarity also explains that other biometrics such as fingerprints [5] or palmprints [6] have been investigated in twins for identification purposes. In the case of forensic phonetics, including twins as participants in research experiments is of interest because these subjects may serve to assess how the results of pairwise comparisons – for the investigated voice characteristics – vary when highly similar speakers are considered (most often identical and fraternal twins but the variability of results can be observed considering also non-twin siblings or singletons). On the other hand, the relevance of twins is closely related with the search for robust voice characteristics for speaker discrimination, since a set of characteristics are considered robust for speaker comparison as far as they are maximally dependent on the speaker’s genetic endowment and minimally influenced by learned factors, the latter favoring voice disguise or imitation. The predominance of genes over environment is thus linked to the two most important criteria for identifying characteristics for Forensic Speaker Comparison (FSC), namely that it should be as consistent as possible for each speaker, i.e. low intra-speaker variability, and that it should exhibit large variation amongst speakers, i.e. high inter-speaker variability [7], [8]. Kinnunen and Li [9] refer to the same characteristics for an ideal Automatic Speaker Recognition (ASR) system.

Several acoustic parameters have been proposed to assess voice similarity in twins, the most common ones being fundamental frequency [10], formant patterns [11], or temporal characteristics [12], although ASR approaches are also common [13], [14]. More recent investigations [3], [15], [16], [17] have focused on the glottal analysis of twins, following a methodology that relies on the decoupling of the vocal tract from the glottal source estimates [18] and which allows the extraction of cepstral coefficients of the glottal source Power Spectral Density (PSD), singularities of the glottal source PSD, biomechanical estimates of vocal fold mass, tension and losses or time-based glottal source coefficients, among others. These features have the advantage of modeling the vocal folds and the vocal tract separately, which opens the possibility of independently studying source and filter information. The approaches in Refs. [3], [15], [16], [17] present a clear advantage as far as the easy extraction of the speech material is concerned. In the cited studies, as well as in the present investigation, the glottal source features are extracted from naturally sustained vowels found in hesitated speech; also known as *fillers* or referred to as *disfluences* by other authors.

The main drawback for conducting more source-related studies in forensic phonetics in the past has been linked to the need for relatively long and stable vocalic sounds from which reliable values of distortion features like jitter and shimmer could be extracted. In clinical settings, these sounds are normally elicited upon asking the subject to sustain a vowel (typically [a]) for as long and steadily as possible [19], [20]. This technique is unrealistic in a forensic context, but previous studies in Spanish suggest that [a] can be replaced by the use of naturally sustained pause fillers (typically [eː] in Spanish; [3]), as they are more forensically realistic while long enough for estimating a sufficient number of glottal cycles. This type of *disfluencies*, which are characteristic of spontaneous speech, have recently become a fruitful area of research interest. Künzel [21] already highlighted the consistency of speakers in their respective use of a personal variant of the hesitation sound, whether in relation to the addition of a bilabial nasal consonant or as regards the specific timbre of the vocalic component.

More recent studies have investigated formant values in filled pauses [22], or have focused on their duration and frequency of occurrence [23]. The extraction of voice quality features from fillers is less common [24]. The current study provides a new perspective to this type of disfluencies by analyzing 309 hybrid acoustic features to test their forensic potential in distinguishing same-speaker and different-speaker comparisons. This includes testing their robustness with very similar-sounding speakers, i.e. identical twins. In addition, this study explores novel methods for measuring (dis)similarities between subjects in pairwise comparisons, such as Euclidean Distances (ED). In twin studies, this type of statistical mapping has been recently used in Refs. [25], [26]. Whereas both make use of ED, the former focuses on non-phonetic aspects (blood plasma lipidomics profiles), and only the latter is a phonetic study (a case study considering just one twin pair). In FSC in particular, French and colleagues [27] have explored ED to measure similarity between non-twin speaker pairs, including scores obtained from perceptual voice evaluations using the Vocal Profile Analysis (VPA) Scheme [28].

## 2 Materials and methods

This section presents the dataset used in the study and describes the methodology used to process the data. In the Section 2.2 we have distinguished between the acoustic analyses and the perceptual assessment of voices.

### 2.1 Data

We have used the phonetic corpus of Spanish male twins and siblings described in Refs. [3], [29]. This comprises 54 speakers recruited ad hoc for the forensic phonetic investigation of twin and non-twin siblings in Spanish. To the best of our knowledge no other voice databases hitherto exist on twin voice research for the North-Central Peninsular Spanish variety. Although the database also includes dizygotic (DZ) twins and non-twin siblings, for this study we have only selected the available MZ twins (24 speakers) – all of the pairs having been raised together – and the group of unrelated speakers (12 speakers). The number of DZ twins was not enough to perform differential analysis; hence these samples were not considered.

Each speaker was recorded on two different occasions, separated by 2–4 weeks, in order to account for within-speaker variability. The two recording sessions took place in the Phonetics Laboratory of the *Consejo Superior de Investigaciones Científicas* (*CSIC*) in Madrid. The speakers were required to come in pairs for the voice recordings: with their co-twin in the case of MZ twins, and with a friend or work colleague in the case of unrelated speakers. This was aimed at attaining a comparable speaking style to what may be expected in conversations between twins, usually characterized by their spontaneity due to their close relationship. The age of the speakers of this database ranged between 18 and 52 years old (median: 28, interquartile range: 10). All participants were native speakers of North-Central Peninsular Spanish (see Ref. [30] for a description of this variety, also known as Standard Peninsular Spanish). A thorough questionnaire completed by all the participants served to assess health habits at the time of the recordings as well as to evaluate the degree of relationship closeness between pairs (only in the case of twins) by using Likert scales and typical questions used in previous phonetic studies on twins [11]. Besides, the zygosity of all the twins was checked; only for a MZ twin pair a DNA testing was necessary, which served to confirm that they were actually MZ twins.

Although the selected twin corpus included several speaking tasks, for this study we have only used the fifth speaking task: informal interview between each speaker and the first author of this investigation (the speaking styles exhibited by the participants were comparable to those found in forensic recordings). The interview lasted approximately 10 min and was carried out on the telephone, i.e. the researcher is at one end of the telephone and one member of each speaker pair at a time is at the other end of the telephone, in a different room. The recordings were made with high-quality but unobtrusive microphones (omnidirectional, condenser and flat-frequency-response microphones in an ear-set device). Forensically realistic conditions were thus achieved, also thanks to the minimization of the *observer*’*s paradox*, well known in sociolinguistic studies [31]. Similar recording scenarios are found in the forensic phonetic literature [32], [33]. In this speaking task the subjects were asked about the topic discussed in the first task. Since there is a considerably long time gap between the first and the fifth task, the speakers do not remember clearly the whole conversation and they exhibit hesitating responses. This gives rise to the so-called *fillers,* a type of speech disfluencies which in Spanish typically take the phonetic form of a long [e], usually transcribed as [eː]. The forensic potential of these naturally sustained vocalic sounds was highlighted in Section 1. For each of the 54 speakers, we extracted 6–10 tokens in each of the two sessions. In total, the dataset used in this study consists of 880 tokens of the [eː] vowel, with an approximate duration of 160 ms. These tokens were manually located and extracted using *Praat* [34]. We excluded five tokens which were very short because it was not easy to perform a robust estimate of some acoustic features on them, thus processing 875 tokens.

The voice data are available in two different qualities. Originally, the data were extracted from high-quality recordings (44,100 Hz sample rate, 16-bit resolution, and mono channel). In a second step, the 875 tokens were band-passed filtered in order to obtain more forensically realistic data, usually characterized by being channel-degraded due to the telephone transmission. To simulate band pass reduction, the voice signal was low-pass filtered at 3.4 kHz, high-pass filtered at 300 Hz and downsampled to 8 kHz.

### 2.2 Methods

This section describes first the acoustic methodology for the processing of the speech tokens, using a range of speech signal processing algorithms (feature extraction). After that, the methodology for the exploratory data analysis and feature selection is described. Before getting into the details of the perceptual analysis, which comes at a second stage, a description of the metric used to calculate speaker similarities is provided.

#### 2.2.1 Acoustic analysis

##### 2.2.1.1 Acoustic characterization of the voice signals (feature extraction)

We applied the 309 speech signal processing algorithms summarized previously in Refs. [35], [36], [20]^1^: these tools were originally developed to process sustained vowel phonations and assess neurological disorders which leave an imprint in voice. These algorithms include traditional perturbation measures such as jitter (f0 variation) and shimmer (amplitude variation): for the algorithmic definition of the various jitter and shimmer variants used, see Ref. [20]. Moreover, many of these tools rely on quantifying signal to noise ratio using a range of algorithmic approaches: these tools include the Glottal-to-Noise Excitation (GNE), the Vocal Fold Excitation Ratio (VFER), and the Empirical Mode Decomposition Excitation Ratio (EMD-ER). Other tools include variability of f0 (e.g. using wavelet-based approaches). The complete list of features appears in Table 1.

**Table 1:** Feature set names, description and number of features per category (total: 309 different features).

Feature	Description	Number of features
Jitter variants	Fundamental frequency perturbations	30
Shimmer variants	Amplitude perturbations	21
Harmonics to noise ratio	Signal to noise ratio using autocorrelation	4
Glottal quotient	Quantifying vocal fold cycle variability	3
Recurrence period density entropy (RPDE)	Uncertainty in estimation of fundamental frequency	1
Detrended fluctuation analysis (DFA)	Stochastic self-similarity of turbulent noise	1
Pitch period entropy (PPE)	Quantifying variability in F0 over and above normal variability in healthy controls	1
Glottal to noise excitation (GNE)	Noise synchronization in different frequency bands	6
Vocal Fold Excitation Ratio (VFER)	Noise synchronization in different frequency bands	9
Empirical Mode Decomposition Excitation Ratio (EMD-ER)	Decomposing the signal in multiple time series using EMD and quantifying energy and entropy	6
Mel Frequency Cepstral Coefficients (MFCC)	Amplitude and spectral fluctuations	42
F0-related measures	f0 statistical characterization, differences compared to age- and gender-matched healthy controls	3
Wavelet-based measures	Characterizing f0 using wavelet decomposition methods	182

Many of the acoustic analysis algorithms reviewed above require the computation of f0 estimates. Recently, Tsanas and colleagues [37] compared 10 well-established f0 estimation algorithms in the speech signal processing literature validating their findings in two databases where the ground truth f0 was known a priori. Moreover, a novel f0 fusion scheme had been proposed which was reportedly leading to consistently more accurate f0 estimates than the individual f0 algorithms. In this study, we use that f0 fusion scheme to obtain the f0 estimates, which were subsequently fed into the acoustic analysis algorithms when required. Finally, we also computed 42 Mel Frequency Cepstral Coefficients (MFCCs), which are one of the most widely-used acoustic analysis methods, with applications in both speech recognition [38] and more recently in speaker identification. MFCCs focus primarily on the articulators (mouth, tongue, lips), and are traditionally used as the standard benchmark in speaker recognition systems against which novel techniques are compared to.

Overall, we characterized each signal in the database using 309 speech signal processing algorithms, resulting in a design matrix of size 875 × 309. There were no missing entries in the design matrix.

##### 2.2.1.2 Exploratory data analysis and feature selection

Exploratory analysis refers to visualizing the data and using formal statistical analysis algorithms to explore certain hypotheses and quantify statistical association strengths. The data was non-Gaussian, and hence we used the Spearman correlation to report association between the features and the outcome (if the speakers are twins or unrelated speakers).

We used the LOGO algorithm to select features [39], and applied the feature selection methodology described in previous studies [40], [41] in order to decide on the feature subset with maximal generalization and predictive ability. In short, that methodology uses perturbed versions of the original dataset (we used 100), selecting features on each of these cases, and then using a voting mechanism to determine the final ranking of the feature set.

##### 2.2.1.3 Euclidean Distances

The metric used to calculate speaker similarity was the Euclidean Distance (ED). This well-known distance measure, also referred to as Pythagorean distance, is commonly defined as the square root of the sum of the squares of the differences between the corresponding coordinates of two points; or simply as the straight-line distance between two points in the Euclidean space. Since the different voice features considered for calculating this distance are on completely different scales of measurement, some form of standardization was necessary to balance out the different contributions of variables and to avoid that any of them dominate in the calculation of the ED. We therefore calculated pairwise distances between speakers using Standardized Euclidean Distances. Variables are thus transformed so they all have the same variance of 1.

(1) $$\begin{array}{c} {d_{St}^{2}=\left(x_{S}-x_{t}\right)V^{-1}\left(x_{S}-x_{t}\right)}^{\prime }\\ \text{S}\text{tand}\text{ardized}\;\;\;\text{Euclidean}\;\;\;\text{D}\text{is}\text{tance} \end{array}$$

Eq. (1) is the formula for calculating the Standardized Euclidean Distance, where V is the n-by-n diagonal matrix whose *j*th diagonal element is s(*j*)^2^, where **s** is the vector of standard deviations.

#### 2.2.2 Perceptual analysis

##### 2.2.2.1 Vocal Profile Analysis Scheme (VPAS)

The Vocal Profile Analysis Scheme (VPAS) is a perceptual approach to the description of voices and more accurately to the analysis of voice quality (VQ). This protocol grew out of some early work by Laver [28], [42] but has evolved to slightly different schemes since its inception as a clinical and research tool for voice analysis. Fuller descriptions can be found in Refs. [43] or [44]. While other perceptual protocols are available for voice analysis (e.g. GRBAS, SVEA, CAPE-V, described in Refs. [45], [46], [47], respectively) their use is more widespread among clinicians; the VPAS being most widely used by phoneticians, and also most popular among forensic practitioners, to a great extent thanks to studies such as Refs. [8], [48], [49], where its use is recommended.

The VPAS is defined as a systematic phonetic framework for the descriptions of a speaker’s VQ, where the term VQ encompasses “all the non-segmental features of speech which characterize an individual’s habitual speech patterns” ([50,p. 294]). The general principles underpinning this scheme are: (1) The whole of the vocal apparatus is considered, i.e. habitual patterns of modifications in the vocal tract (e.g. lips, jaw or tongue configurations) contribute as much to an individual’s VQ as habitual configurations of the larynx, i.e the auditory coloring of a speaker’s characteristic voice stemming from phonation modifications; (2) VQ is analyzed in terms of a number of strands, or components, which may be combined in a variety of ways. The term ‘setting’ is used to refer to these components and is defined as a long-term tendency for some part of the vocal apparatus to adopt a particular configuration [51]. A variable number of settings exist depending on the version of the protocol; 36 settings in Ref. [44]; (3) All voices are compared to a ‘neutral setting’, a clearly defined baseline with concrete acoustic and physiological correlates. Deviations from neutral are quantified in a 1–6 degree scale, where 1–3 are classed as ‘moderate’ and 4–6 are classed as ‘extreme’ (cf. [44], [50]).

For our study we have attempted a simplification of the VPAS with a considerable reduction of settings and no scalar degrees, which has enabled us to obtain a simplified method for calculating measures of (dis)similarity between pairs of speakers, as we will explain below in some more detail. The main reason why a simplification of the dimensions/settings of the original VPAS has been deemed necessary for FSC is the high multidimensionality of VQ: while auditory judgments are predicated on the assumption that listeners have a common understanding of perceptual labels [52], perceptual dimensions often overlap and listeners cannot always isolate for judgment one perceptual dimension from several co-occurring dimensions [52], [53]; cf. [54]. This justified the simplification and merger of very similar settings in the VPAS, for the sake of improving the reliability and validity of this methodology in a forensic scenario.

The main characteristics of the Simplified VPAS (SVPAS) suggested here for FSC are as follows:

1. There are 10 ‘major setting groups’ and a total of 26 possible settings within those 10 groups (see Table 2); the intermittent presence of a setting is no longer marked. Instead of using scalar degrees 1–6, it is proposed that for each setting the rater simply marks whether the voice is *neutral* (i.e. absence of a remarkable deviation) or *non-neutral* (i.e. presence of a remarkable deviation).

**Table 2:** Simplified Vocal Profile Analysis Scheme (SVPAS). Full names of the abbreviations used in the table: Mandib.: Mandibular; Ling.: Lingual; Pharyng.: Pharyngeal; Velo-pharyng.: Velo-pharyngeal; VT: Vocal Tract; L: Laryngeal; Phon.: Phonation; Labiodent.: Labiodentalization; Protr.: Protruded; Creak.: Creakiness.; Whisp.: Whisperiness.

Key	Major setting groups									
	Labial	Mandib.	Ling. tip	Ling. body	Pharyng.	Velo-pharyng.	Larynx height	VT tension	L tension	Phon. types
1a	Lip rounding	Close	Advanced	Front & raised	Constricted	Audible nasal escape	Raised larynx	Tense	Tense	Falsetto
1b	Lip spreading	Open	Retracted	Back & lowered	Expanded	Nasal	Lowered larynx	Lax	Lax	Creak.
1c	Labiodent.	Protr.				Denasal				Whisp.
1d										Harsh.
1e										Tremor
2. If the voice is considered neutral for a specific setting, a 0 is assigned in a table such as the one presented in Table 3. If non-neutral, a decision should be taken on the direction of the deviation from neutrality.

**Table 3:** Example of calculation of Simple Matching Coefficients (SMC) for MZ twin pair 41–42.

		Major setting groups										
		Labial	Mandib.	Ling. tip	Ling. body	Pharyng.	Velo-pharyng.	Larynx height	VT tension	L tension	Phon. types	
Speakers	41	0	1a	1a	0	0	0	0	1b	1b	1c	
	42	0	1a	0	0	0	0	1b	1b	1b	1c	
Matches		1	1	0	1	1	1	0	1	1	1	0.8
												SMC
3. For most setting groups (e.g. lingual tip, larynx height, vocal tract tension), only two directions are possible as deviations from neutrality. For instance, for lingual tip: either advanced (1a) or retracted (1b). For other setting groups, however, the possibilities for non-neutrality include up to 5 decisions. See group ‘voicing type’. Therefore, these possibilities need to be expressed as categories (*a*, *b*, *c*, *d*, or *e*). It is no longer a question of absence or presence of neutrality, but if non-neutrality is perceived for a category (e.g. absence of labial neutrality), a decision has to be taken on the direction of the non-neutrality (e.g. lip rounding).
4. Besides, there is no marking following anatomical progression down the vocal tract from the lips to the larynx, as suggested in Ref. [44]. Instead, the category labeling is carried out by marking first what is more remarkable for the rater and then trying to decide on the rest of major settings.

The main modifications toward simplification of the original VPAS can be summarized as: reduction from 36 settings to 26 and no use of scalar degrees. Besides, within each major setting group, a decision has to be taken as regards the direction of the deviation from neutrality, while in the original protocol it is possible to select several options. For instance, in relation to voicing type, a rater (i.e. the analyst, or expert who gives a rating to the voice) could label a voice with *creakiness* and *harshness*. While it is well known that there are combined phonation types, usually one is predominant – which is the one that has to be rated in our SVPAS – and the other/s only appear intermittently. For most of the remaining major settings, our simplified rating system is perfectly apt to the mutually exclusive nature of labels: e.g. in relation to the vocal tract (VT) tension, if the speaker is non-neutral for that setting, he presents either tense VT or lax VT; or if he is non-neutral as concerns the lingual body, he will either tend to present a fronted and raised tongue body or a backed and lowered tongue body. The main modifications from the original settings have been made for phonation types. We are no longer distinguishing between subgroups ‘voicing type’, ‘laryngeal frication’ and ‘laryngeal irregularity’. All of them are merged now into phonation types. Furthermore, for the sake of simplification – and because the boundaries are sometimes blurred – there is no distinction between creak and creaky and whisper and whispery, as in the VPA version described in Ref. [44].

##### 2.2.2.2 Simple Matching Coefficients (SMC)

The simplification of the VPA protocol has been envisaged in order to obtain a numerical measure of the distance between two speakers using ED for perceptual evaluations, which could then be compared with the ED calculated for acoustic features. Considering that ED for categorical data are best computed using a Simple Matching Coefficient (SMC) method, we will explain below how this technique was implemented for our data.

If only one variable existed (for instance, labial setting), computing the distance between two speakers would be fairly trivial: imagine two speakers have the same configuration for that setting (e.g. lip rounding); their distance would be 0. If one of them had lip rounding and the other lip spreading, their distance would be 1. Also, if one of them was neutral for that setting and the other had any type of deviation from neutrality – in this case, either lip rounding or lip spreading – the distance would be 1 as well. As not only one but several categorical variables (labial setting, mandibular setting, etc.) exist for calculating the distance between two speakers, the simplest method is that of extending the ‘matching’ idea and counting how many matches and mismatches there are between samples. In the case shown in Table 3, there are 8 matches and 2 mismatches between speakers 41 and 42, hence the distance between the two speakers is 8 divided by 10, the number of variables, that is 0.8. This is called the Simple Matching Coefficient (SMC).

A total of 29 speakers (24 MZ speakers and 5 speakers pertaining to the DS group) were perceptually evaluated using the SVPAS by listening to recordings of spontaneous speech samples (90–120 s) of each speaker. These speech samples were extracted from the corpus described in Ref. [29] (same speaking task from where the pause fillers were extracted for the acoustic analysis). The voices were listened by the first author at least twice before completing the SVPAS protocol. Then ED in the form of SMC were calculated for the 12 MZ pairs and three further DS pairs, corresponding to the speakers with higher ED in the HQ condition. Our aim here was to find how the ED in the acoustic domain correlate with ED found in the perceptual domain.

## 3 Results

### 3.1 Pre-test: stability of f0 contours in pause fillers

**Fig. 1:**
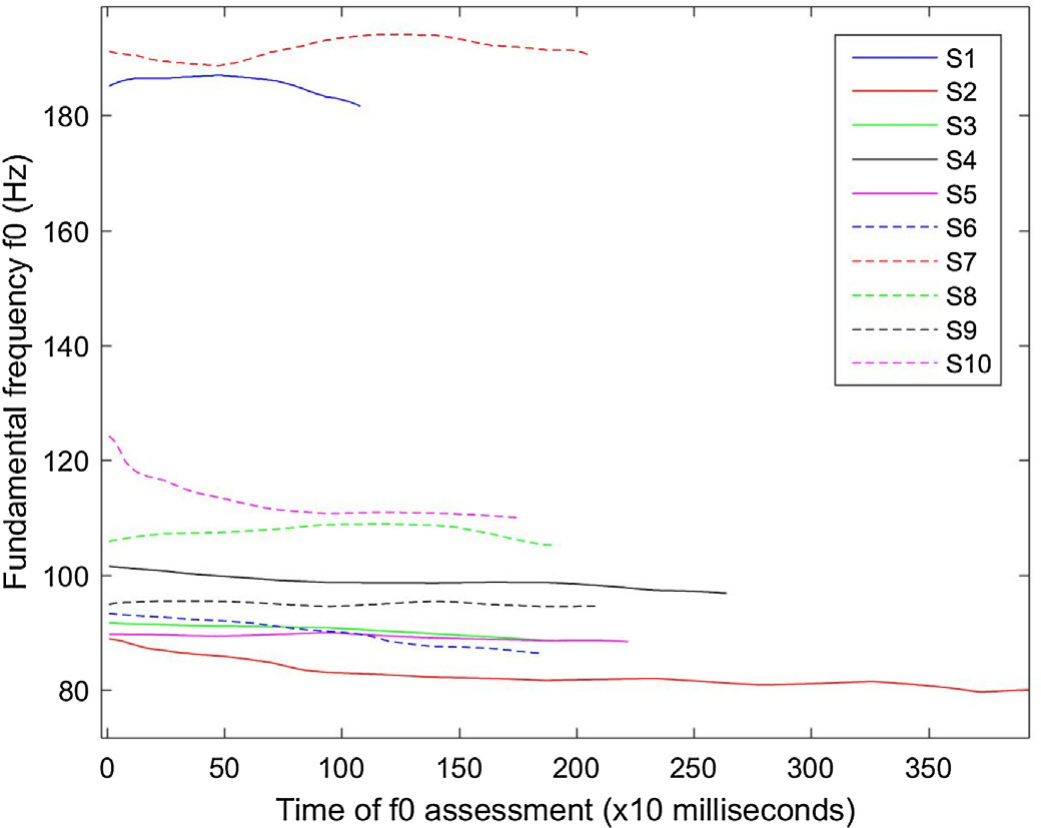
Fundamental frequency (f0) contour of 10 randomly selected tokens to visually assess f0 variability. Each token corresponds to different subjects (S).

As a first step before any analysis, we tested the previously reported observation [3] that the naturally sustained pause filler [eː] is similar to an artificially sustained vowel, such as long [a], produced in typical clinical studies, for instance, to calculate maximum phonation time. Firstly, visual inspection allowed us to verify that the pause fillers were actually “stable”, i.e. the amplitude and frequency remain relatively constant. To objectively assess this we computed the f0 contours: we expect to see f0 contours which are relatively stable (or at least stable over a certain time window), exhibiting fluctuations like those observed in sustained vowels. Visual inspection of these contours corroborated previous reports in the research literature that the pause fillers can be considered sufficiently stable, at least during the middle of the pause filler. Fig. 1 presents some randomly selected samples to demonstrate this.

### 3.2 Acoustic analysis

#### 3.2.1 Euclidean Distances

Feature selection methodology determined the most parsimonious feature subset comprising the 15 jointly most statistical predictive features. Standardized Euclidean Distances (ED) were then calculated using those features for all the possible paired speaker combinations in our database (54 × 54). As there are only 54 possible same-speaker (SS) comparisons, the number of different-speaker (DS) comparisons was reduced also to 54. These DS pairings were randomly selected considering the following arithmetic progression: e.g. speaker 1 with speaker 3, speaker 2 with speaker 4, and so on. The total number of MZ pairs in the database was 12. As can be seen in Table 4, speakers 1–11 are paired with speakers 2–12; speakers 33–43 are paired with speakers 34–44. Table 5, Table 6 summarize the ED for SS, DS, respectively, distinguishing between high quality (HQ) condition and telephone-filtered (TF) condition.

**Table 4:** ED values in high-quality (HQ) and telephone-filtered (TF) condition for the 12 MZ pairs. The values considered outliers are shown in italics, corresponding to the strongest dissimilarity (pair 11–12 for both conditions; pair 35–36 for TF condition).

SP_1	1	3	5	7	9	11	33	35	37	39	41	43
SP_2	2	4	6	8	10	12	34	36	38	40	42	44
HQ	6.11	6.86	6.54	8.18	6.27	*16.19*	6.04	6.16	5.95	7.26	5.44	6.43
TF	6.37	7.22	6.91	8.64	10.70	*83.07*	6.00	*21.58*	5.48	10.49	6.11	6.96

**Table 5:** ED values in high-quality (HQ) and telephone-filtered (TF) condition for the 54 same-speaker comparisons.

SP_1	1	2	3	4	5	6	7	8	9	10	11	12	13	14	15	16	17	18
SP_2	1	2	3	4	5	6	7	8	9	10	11	12	13	14	15	16	17	18
HQ	5.32	5.34	5.38	5.34	5.41	5.33	5.39	5.35	5.34	5.32	5.33	5.31	5.36	5.33	5.40	5.41	5.26	5.37
TF	5.31	5.30	5.30	5.35	5.36	5.19	5.35	5.31	5.27	5.30	5.31	5.26	5.28	5.31	5.36	5.31	5.23	5.32

SP_1	19	20	21	22	23	24	25	26	27	28	29	30	31	32	33	34	35	36
SP_2	19	20	21	22	23	24	25	26	27	28	29	30	31	32	33	34	35	36
HQ	5.35	5.39	5.35	5.30	5.38	5.35	5.43	5.35	5.33	5.34	5.35	5.38	5.37	5.33	5.39	5.36	5.41	5.33
TF	5.32	5.34	5.28	5.16	5.30	5.23	5.36	5.24	5.27	5.28	5.36	5.26	5.30	5.32	5.37	5.33	5.37	5.33

SP_1	37	38	39	40	41	42	43	44	45	46	47	48	49	50	51	52	53	54
SP_2	37	38	39	40	41	42	43	44	45	46	47	48	49	50	51	52	53	54
HQ	5.31	5.36	5.35	5.41	5.41	5.40	5.35	5.35	5.34	5.28	5.27	5.36	5.34	5.34	5.36	5.38	5.39	5.37
TF	5.33	5.34	5.34	5.40	5.36	5.33	5.28	5.34	5.25	5.20	5.22	5.29	5.34	5.33	5.34	5.33	5.31	5.36

**Table 6:** ED values in high-quality (HQ) and telephone-filtered (TF) condition for the 54 different-speaker (DS) comparisons. The values considered outliers are shown in italics, corresponding to the strongest between-speaker dissimilarity. The values in bold are the lowest ED for DS comparisons, and they overlap with the average ED for SS comparisons (False acceptances).

SP_1	1	2	3	4	5	6	7	8	9	10	11	12	13	14	15	16	17	18
SP_2	3	4	5	6	7	8	9	10	11	12	13	14	15	16	17	18	19	20
HQ	11.44	6.85	9.06	7.99	7.16	10.01	13.36	15.69	8.55	8.64	*138.6*	9.77	8.16	7.15	10.41	11.49	9.26	9.14
TF	*33.33*	6.87	7.13	6.71	8.11	7.50	11.78	18.17	8.76	8.67	*44.90*	7.30	6.97	7.01	8.52	7.24	32.87	7.68

SP_1	19	20	21	22	23	24	25	26	27	28	29	30	31	32	33	34	35	36
SP_2	21	22	23	24	25	26	27	28	29	30	31	32	33	34	35	36	37	38
HQ	7.64	*282.2*	8.53	8.93	7.61	6.94	7.86	10.53	19.41	9.07	8.20	7.90	7.68	7.93	6.88	7.14	8.15	6.06
TF	6.61	17.83	6.89	**5.34**	11.56	7.27	7.68	8.02	6.40	13.97	9.77	7.72	6.49	9.89	7.19	*51.20*	6.42	**5.15**

SP_1	37	38	39	40	41	42	43	44	45	46	47	48	49	50	51	52	53	54
SP_2	39	40	41	42	43	44	45	46	47	48	49	50	51	52	53	54	1	2
HQ	10.73	15.03	8.00	15.66	6.62	6.70	7.88	31.37	*222.9*	6.09	6.17	9.05	6.90	7.49	6.53	13.82	7.31	8.80
TF	9.35	19.19	9.02	11.92	6.21	6.41	10.36	21.14	20.36	4.59	6.10	9.17	5.90	7.29	**5.65**	8.91	8.39	6.37

**Fig. 2:**
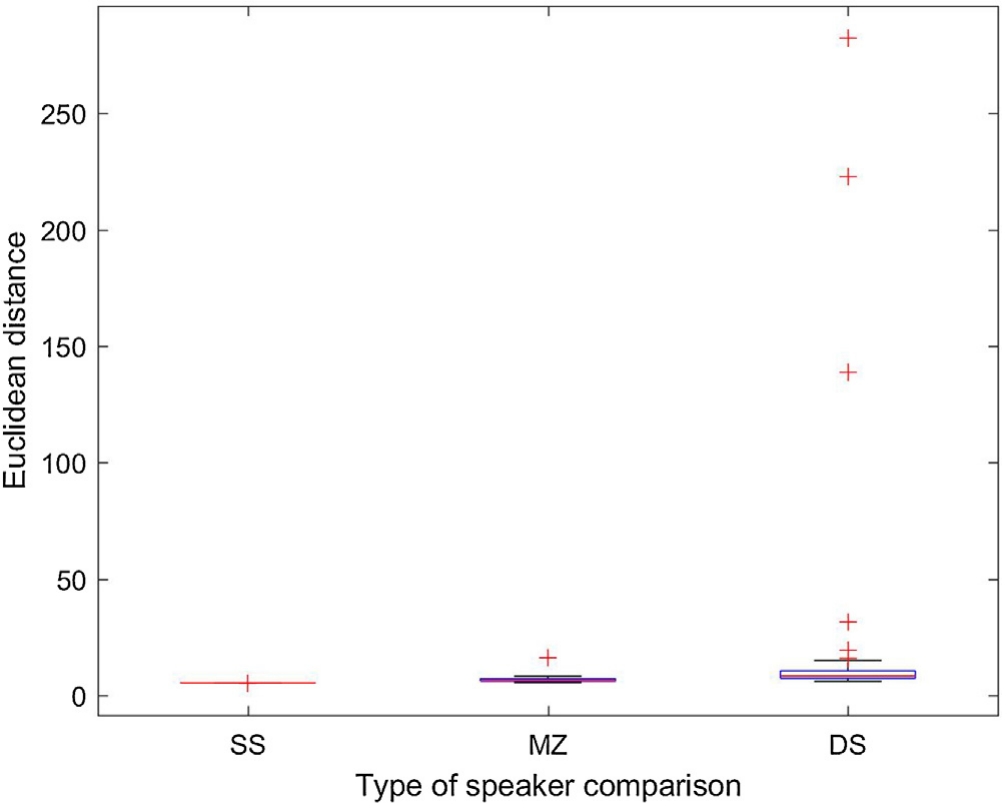
ED distribution per type of speaker comparison (SS: same speaker; DS: different speakers; MZ: monozygotic pairs) in the high quality (HQ) condition.

**Fig. 3:**
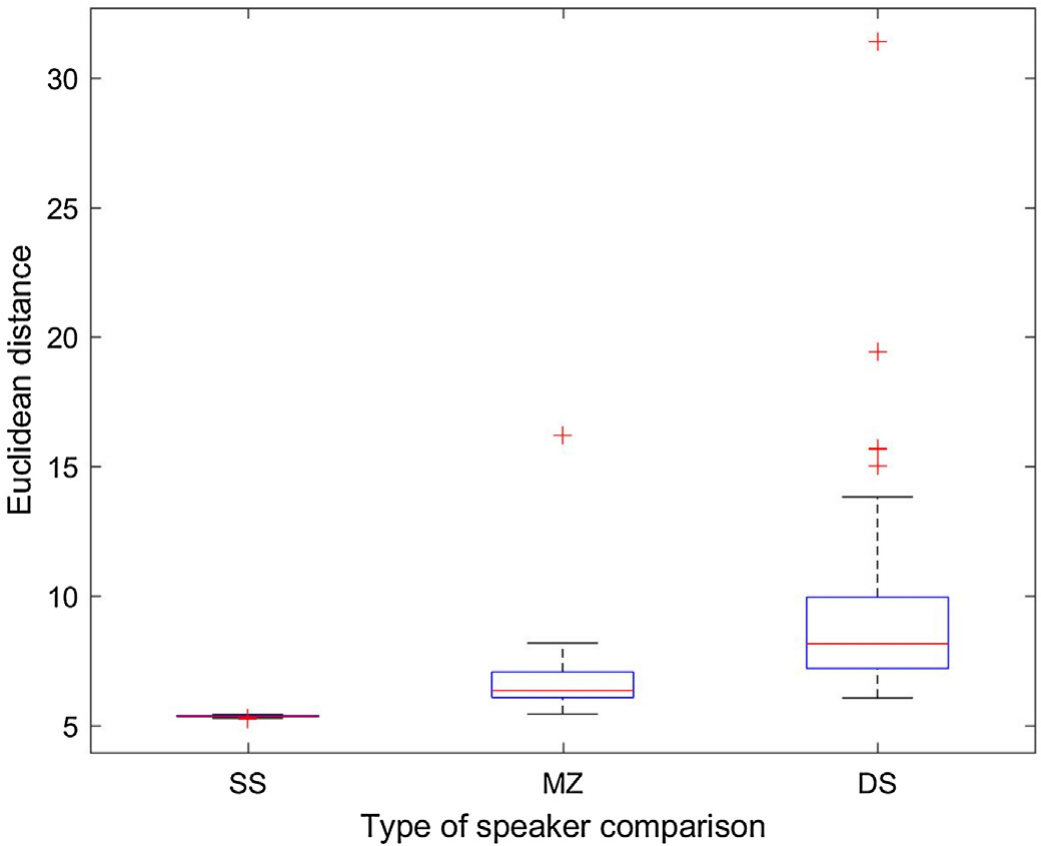
ED distribution per type of speaker comparison (SS: same speaker; DS: different speakers; MZ: monozygotic pairs) in the high quality (HQ) condition: zoom view after removing the three outliers in Fig. 2, i.e. speaker pairs 11–13, 20–22 and 45–47.

Fig. 2, Fig. 3 present the distribution of ED per type of speaker pairing, only for the HQ condition. The boxplots show that the ED for the same-speaker comparison (n = 54) are very homogenously distributed (mean: 5.35; standard deviation: 0.04). See also Table 5 where none of the ED values outstands among the others as an outlier.

In the case of MZ pairs (n = 12), ED values are also quite evenly distributed with a mean higher than for SS comparisons, indicating a slightly higher dissimilarity between the speakers compared (mean: 7.29; standard deviation: 2.89). Only one MZ pair (11–12) could be considered an outlier with an ED of 16.19 (see Table 4), indicating stronger dissimilarity than for the average MZ pair.

Finally, in the case of DS comparisons (n = 54), ED values gather around 20 but their distribution is far from even (mean: 20.90; standard deviation: 49.61). Mainly three DS pairs outstand as strikingly dissimilar. Upon looking at Table 6, we find that these pairs are 11–13, 20–22 and 45–47 with ED values of 138.6, 282.2 and 222.9, respectively. Pairwise Wilcoxon ranksum tests showed that the differences between all three groups (SS, DS and MZ) are statistically significant (p < 0.001).

**Fig. 4:**
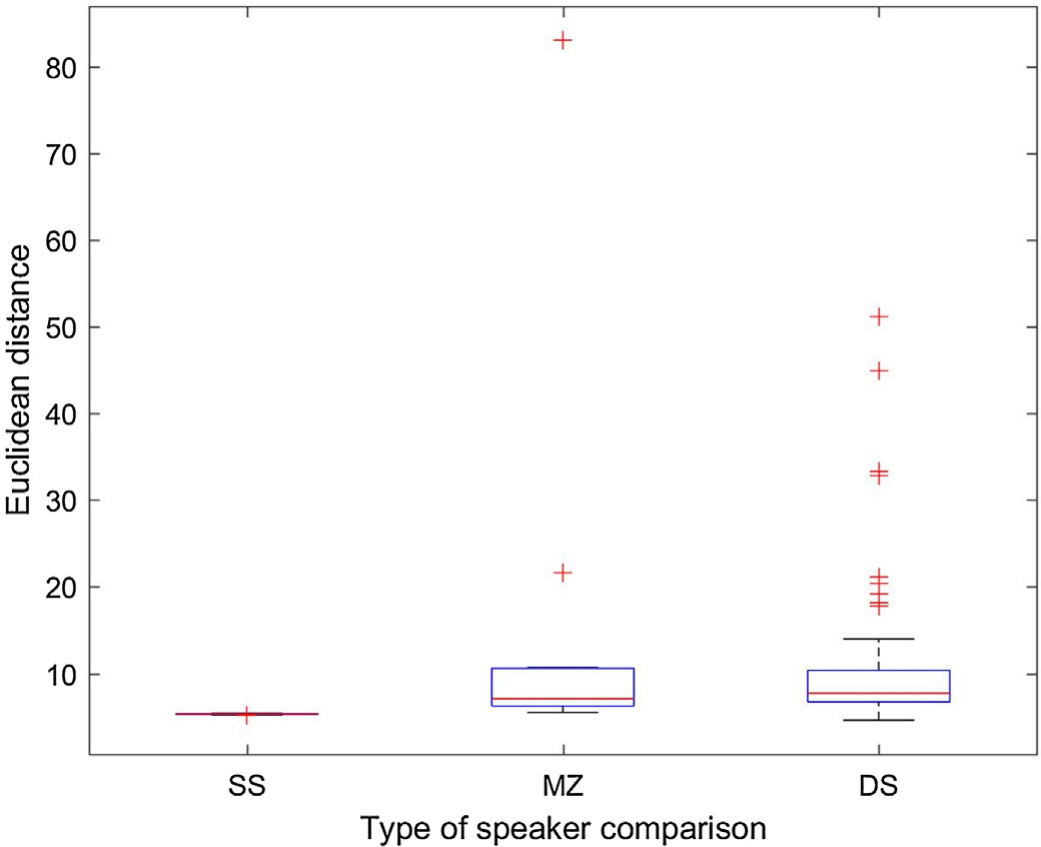
ED distribution per type of speaker comparison (SS: same speaker; DS: different speakers; MZ: monozygotic pairs) in the telephone-filtered (TF) condition.

Fig. 4 presents the distribution of ED per type of speaker pairing, this time for the TF condition. The boxplots show that the ED for the same-speaker comparisons (n = 54) are again very homogenously distributed (mean: 5.31; standard deviation: 0.05), with values very similar to those found in the HQ condition.

In the case of MZ pairs (n = 12), ED values present a higher mean than for SS comparisons, as in the HQ condition, indicating higher dissimilarity between speakers, although in this case the standard deviation is much higher (mean: 14.96; standard deviation: 21.89). If in the HQ condition only one MZ pair was detected as an outlier (11–12), in the TF condition we still find pair 11–12 with an strikingly high ED (83.07) but another outlier value appears in the boxplot (21.58), corresponding to MZ pair 35–36. Indeed, MZ pair 11–13 turns out to be more dissimilar than the most dissimilar DS pair. The striking difference found for this identical twin pair agrees with previous studies using this same twin database but a likelihood-ratio approach [3]. Possible explanations for this are suggested in Section 4.

Finally, in the case of DS comparisons (n = 54), ED values are not completely normally distributed, as it happened in the HQ condition (mean: 11.32; standard deviation: 9.43), but in comparison with the HQ condition the standard deviation is not so high. Besides, it seems that one of the effects exerted by the telephone filter is that the outlier pairs do not exhibit such high ED. Compare the values 282.2, 222.9 and 138.6 obtained by the three more dissimilar pairs in the HQ condition with the values 51.20, 44.90, 33.33, and 32.87 obtained by the four more dissimilar pairs in the TF condition (see Table 6). Interestingly, only the pair 11–13, outlier in the HQ condition, remains an outlier in the TF condition. In addition, three new outliers (high ED) emerge in the TF condition, corresponding to speakers who were not so dissimilar in the HQ condition: 1–3, 17–19, and 34–36. In contrast, pairs 20–22 and 45–47, with high ED in the HQ condition, show ED aligned with the mean in the TF condition. As in the HQ condition, pairwise Wilcoxon ranksum tests showed that the differences between all three groups (SS, DS and MZ) are statistically significant (p < 0.001).

#### 3.2.2 Z-score normalization

**Fig. 5:**
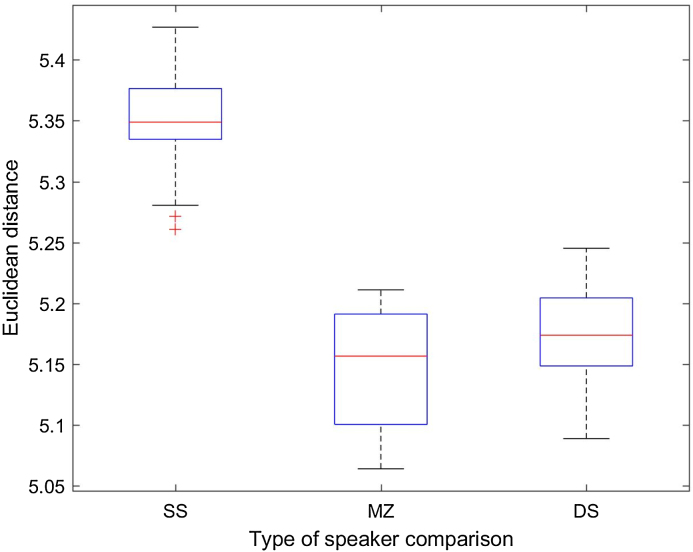
ED distribution per type of speaker comparison (SS: same speaker; DS: different speakers; MZ: monozygotic pairs) in the high-quality (HQ) condition with z-score normalization.

**Fig. 6:**
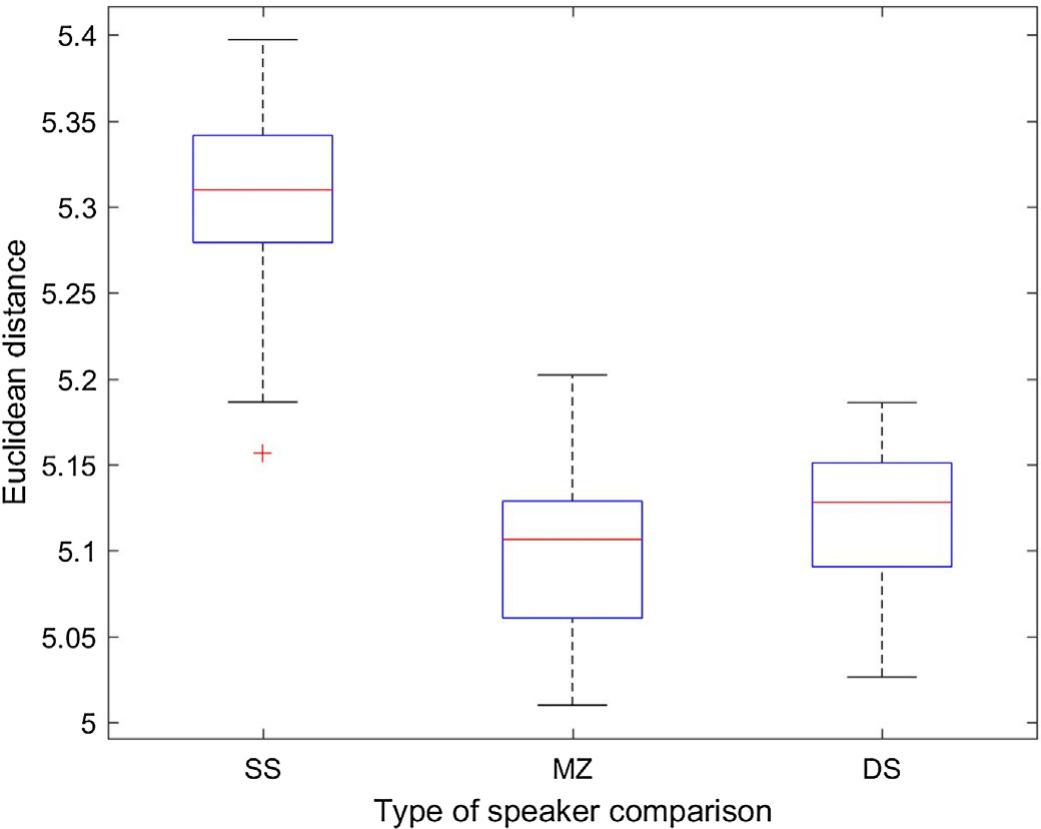
ED distribution per type of speaker comparison (SS: same speaker; DS: different speakers; MZ: monozygotic pairs) in the telephone-filtered (TF) condition with z-score normalization.

In the previous section we have described the method to calculate ED between speaker pairs as a quantitative procedure to measure their similarity. Since each pairwise comparison is based on 15 variables (15 voice features, comprising vocal-tract and laryngeal characteristics), it was expected that some of them contributed more than others to the ED value. Besides, these variables are on completely different scales of measurement. Therefore, some form of standardization was necessary to balance out the contribution of the most dominant variables, so that they do not overshadow in the calculation of the ED. The conventional way to do this is called standardization. An alternative way to do this is to normalize the data of each of the speakers being compared in each ED calculation. For that purpose, we used z-score normalization. Fig. 5, Fig. 6 show the boxplot distribution of SS, DS and MZ comparisons in HQ and TF condition, respectively.

Fig. 5, Fig. 6 show that standardizing the variables to their z-scores has primarily affected MZ and DS comparisons. The distribution for SS comparison was quasi-Gaussian already using the other method (standardized ED). What sees worth highlighting at this point is that with z-score normalization we lose some valuable information which was present when we used the other standardization method. We refer to the detection of the outliers in MZ and DS comparisons. From a forensic phonetic perspective, if we have access to this type of information we can gain relevant insight into the causes of the ED values above the mean in specific MZ pairs. At the same time, if we are able to detect strikingly dissimilar DS, we can discuss the role of these speakers in relation to the biometric menagerie [55], [56] and how they can impair a forensic comparison system, as we will explain in next section.

Likewise, the use of the standardized ED method in comparison with the normalization by z-scores allows us to better assess the effect of the telephone filter. As it was shown in Table 4 (MZ subjects) and especially in Table 6 (DS subjects), and in their corresponding boxplots, different outliers (high ED, stronger dissimilarity) can be detected depending on the condition of the recording. Although in general the TF seems to reduce the ED values of the most dissimilar DS pair, new high dissimilar DS pairs appear who did not outstand as highly dissimilar in HQ condition (see Table 6; pairs 1–3, 17–19, and 34–36).

Most importantly, however, the results obtained using standardized ED and z-score normalization are different. While the use of the former shows that ED are higher for DS pairs and lower for SS comparisons, with the ED values for MZ pairs in between, the results obtained using the second method are against expectations. We find higher ED for SS comparisons, followed by DS pairs and by MZ pairs. This suggests that the first standardization method should be preferred over the z-score normalization.

#### 3.2.3 Heat maps: hunting for *phantoms*

Users do not perform equally well in biometric identification systems. In terms of error rates, two common misclassifications are *false acceptances* and *false rejections*, and the following performance metrics are used: False match rate (FMR, or false accept rate, FAR) is the probability that the system incorrectly matches the input user to a non-matching user in the database, and false non-match rate (FNMR, or False Reject Rate, FRR) is the probability that the system fails to detect a match between the input user and a matching template in the database, thus measuring the percentage of valid matches that are incorrectly rejected. Different combinations of excessive false accepts or rejects exist in biometric systems, so different user groups have been identified in relation to how their performance affects misclassifications or errors [57]. These problem user groups have been given animal names such as *lambs* and *goats* that pretend to analogously reflect the behavior of the denizens of such *biometric zoo* or *menagerie*. First formalized by Doddington and colleagues [55], the original members of this zoo are:

• Sheep: users who produce a biometric that matches well to other biometrics of themselves and poorly to those of other people, i.e. the similarity score is high for genuine comparisons and low for imposter comparisons. Therefore, sheep generate fewer false accepts and rejects than average. These users make up the majority of the population of a biometric system.
• Goats: users who produce a biometric that matches poorly to other biometrics of themselves, i.e. they obtain low similarity scores for genuine comparisons. Therefore, these users are the main responsible for false rejects.
• Lambs: users who produce a biometric that matches well to the biometric of a different person, i.e. they are easy to imitate (by *wolves*); they obtain relatively high similarity scores for imposter comparisons, leading to false accepts.
• Wolves: users particularly good at impersonating other users (*lambs*); i.e. they obtain relatively high similarity scores for imposter comparisons between them and *lambs*. In other words, a wolf has an above average chance of generating a relatively high match score when compared to a stored biometric of a different person [58].

In the biometric menagerie of Yager and Dunstone [56], a revisited version of Doddington’s zoo, four new user groups are proposed (*worms*, *doves*, *chamaleons*, and *phantoms*), defined in terms of a relationship between genuine and imposter match scores. The name *phantom* is then used to refer to those speakers who match poorly against everyone, i.e. they show low match scores regardless of whom they are matched against. For this reason they rarely lead to a false acceptance. Although this would be generally considered positive for a speaker recognition system, this type of users tend to be very different to everyone, including themselves – there is some overlap with goat-like users in the original zoo – so it remains to be fully explored the causes of their inherent “unmatchability” (see Section 4).

**Fig. 7:**
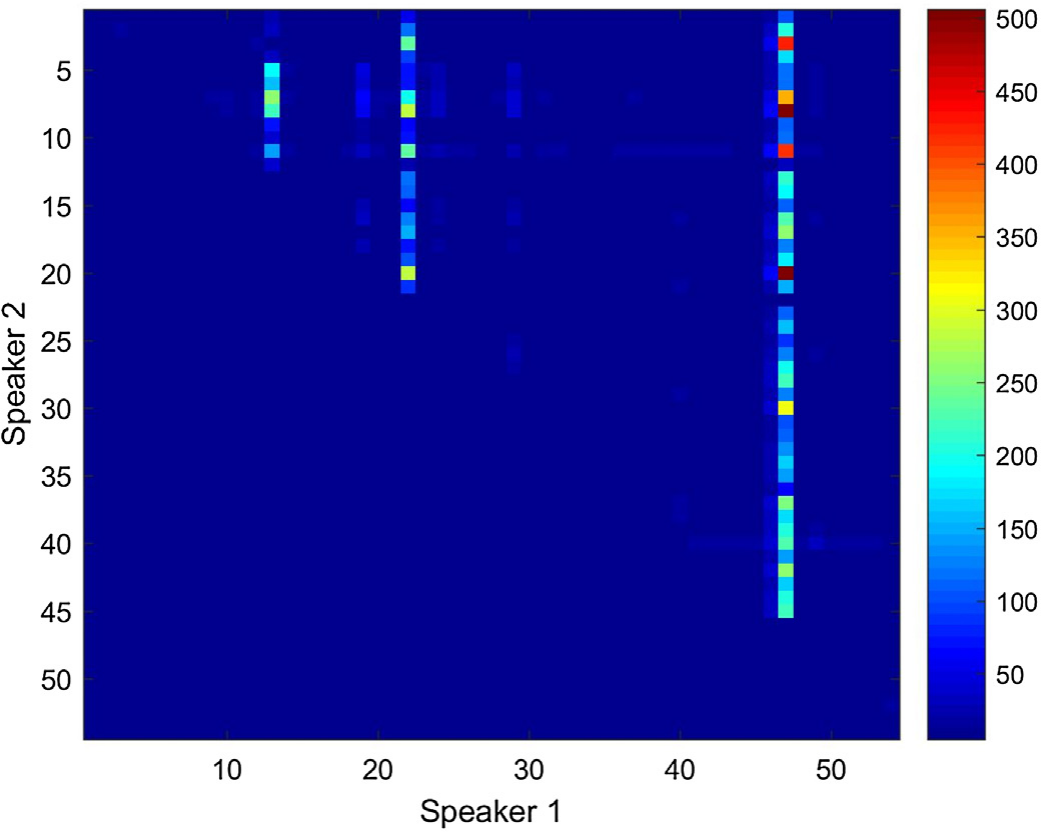
Heat map for all 54 DS comparisons in HQ condition using standardized ED (color bar on the right).

**Fig. 8:**
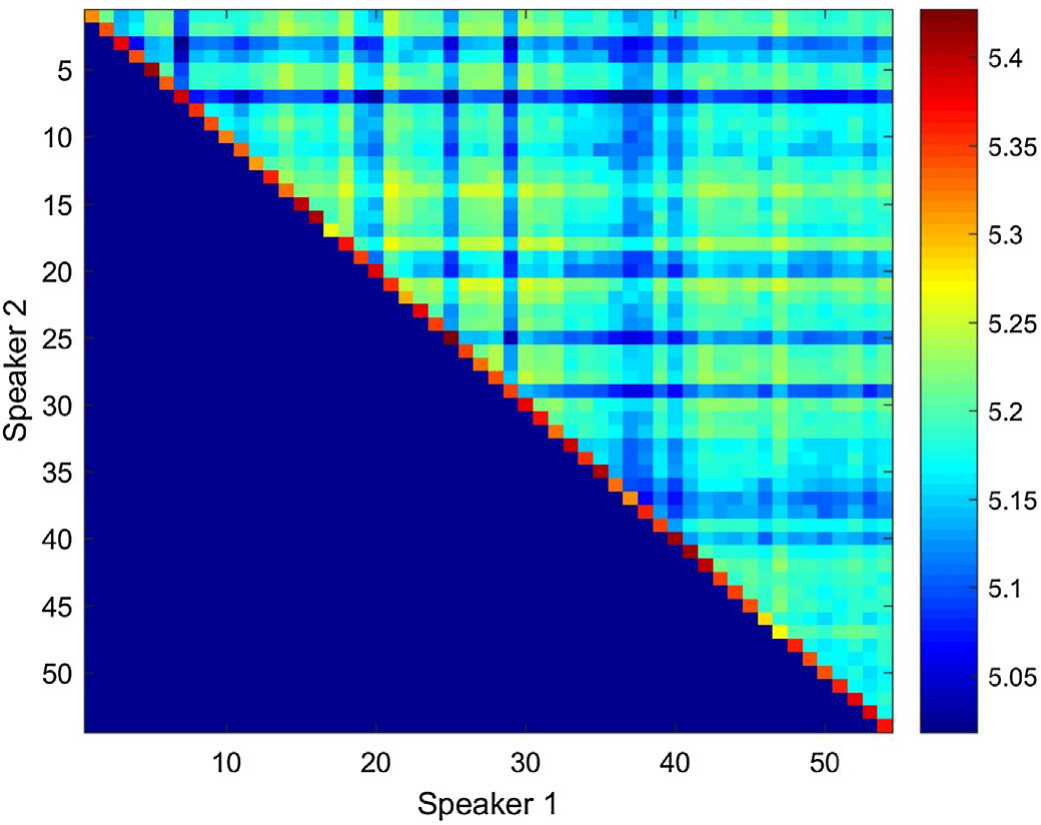
Heat map for all 54 DS comparisons in HQ condition using z-score normalization.

More importantly, if these phantoms are thus deemed to belong to a different population, future work should investigate how the elimination of this type of speakers could help establish a more homogenous database for attaining more robust results. In other words, how would the inclusion of many phantoms in a reference population would affect the likelihood ratio (LR) obtained when comparing a suspect and an offender? Recent studies in this field have investigated similar sources of variability in the analyst decisions during the computation of numerical LRs [59] but more investigations seem necessary in this respect. Heat maps could prove a good visualization technique to detect the so-called *phantoms*. In Fig. 7 this type of plot shows that ED are strikingly high for three main speakers: 13, 22 and 47, irrespective of whom they are being compared with. This information is missing with the z-score normalization technique (Fig. 8). Speaker 47 presents the most phantom-like behavior. In Fig. 7, we show the maximum ED value (499.2) found when comparing his voice with that of a different speaker. All other ED values, typically above 50, are still outliers if we compare them with the average values obtained in DS comparisons (Table 6).

#### 3.2.4 System performance

**Fig. 9:**
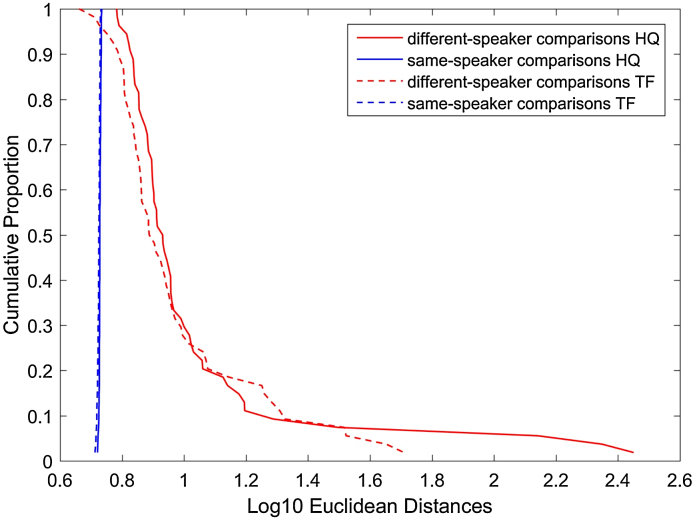
Cumulative proportion of Euclidean Distances (Log 10) for different-speaker (DS) and same-speaker (SS) comparisons. Red lines represent DS pairs while blue lines are used for SS comparisons. Continuous lines depict high quality (HQ) conditions whereas dotted lines are used for telephone-filter (TF) conditions. (For interpretation of the references to color in this figure legend, the reader is referred to the web version of this article.)

Tippett plots are typically used to evaluate the performance of a forensic recognition system. In this type of graph two curves are displayed, each one representing the probability for one of the competing hypothesis: that of the prosecution (Hp) and that of the defense (Hd). Usually the hypothesis of the prosecution is that the offender and the suspect samples come from the same speaker, while the hypothesis of the defense is that they belong to different speakers. Strictly speaking, Tippett plots represent graphically LR- based outputs. Here, we used the ED as a means to visualize the discrimination of the system using inverted cumulative distributions of ED, and not to represent the strength of the evidence. Note that in Tippett plots the strength of the evidence for H0 increases with the log value. In Fig. 9, however, we have not aimed to represent the strength of the evidence since ED only represent the similarity term and not the typicality term of a likelihood ratio (LR). Fig. 9 shows the cumulative distribution of ED (log 10) for the DS comparisons (red lines) and the SS comparisons (blue lines). Some overlap occurs between red and blue lines in the TF condition (dotted lines) while no overlap is observed in the HQ condition. The implication of this is that no false acceptances or missed hits are obtained with an ED-based system in the HQ condition. The same system generates errors in the TF condition because, while SS comparisons still gather around 5.3 (0.7 in log 10), the ED values for some DS pairs are in that margin, or even lower. These cases represent false acceptances.

Besides, the effect of the telephone is obvious in the DS comparisons. The range of values is greater in the HQ condition (with log 10 ED of up to 2.4 in some pairs) than in the TF condition (maximum log 10 being 1.7). This seems in agreement with the fact that the type of bandpass filter occurring in telephone transmissions leaves less spectral information available in the acoustic signal, in comparison with high quality recordings. The acoustic frequencies where speakers may differ are considerably reduced, therefore.

### 3.3 Perceptual analysis

Twenty-nine speakers (24 MZ and 5 DS) were perceptually evaluated using the SVPAS explained in the Section 2. After completing the assessment, ED between pairs of speakers were calculated as SMC. The similarity values obtain range between 0 (very different) and 1 (very similar). A total of 15 SMC were calculated, corresponding to the 12 MZ pairs and three further DS pairs. The latter are the speaker pairs who obtained the highest ED in the acoustic analysis (HQ condition). In other words, in the acoustic domain they were found to be very different. These are used as control subjects in order to observe whether they would also be found very dissimilar applying only a perceptual assessment protocol.

Table 7 shows the ED for the 15 speaker pairs mentioned above. For comparative purposes, the SMC are shown together with the standardized ED obtained by the same pairs in the acoustic analysis. Higher values in acoustic ED means greater dissimilarity while higher values in perceptual ED means greater similarity. In the acoustic domain, the MZ pair 11–12 outstands as very dissimilar (ED = 16.19) in comparison with the rest of identical twin pairs, presenting values homogenously distributed (mean: 7.29; standard deviation: 2.89; n = 12). From a perceptual point of view, differences between twin pairs are not so marked (mean 0.58, standard deviation 0.18; n = 12) and, more importantly, MZ pair 11–12 does not outstand as different from the MZ group. It is still the MZ pair with less perceived differences in voice quality (VQ), but other pair (37–38) also receives a SMC of 0.3. This value indicates that these twins resemble each other in only three out of ten VQ components, while the average trend in MZ pairs is to present perceptual similarities in more than half of their VQ settings.

**Table 7:** Euclidean Distances (ED) between pairs of speakers: monozygotic (MZ) pairs and different-speaker (DS) pairs. Both acoustic ED and perceptual ED are based on high-quality recordings. Perceptual ED are calculated as Similarity Matching Coefficients (MFCs). Higher values in acoustic ED means greater dissimilarity while higher values in perceptual ED mean greater similarity.

	MZ pairs												DS pairs		
Speaker_1	1	3	5	7	9	11	33	35	37	39	41	43	11	20	45
Speaker_2	2	4	6	8	10	12	34	36	38	40	42	44	13	22	47
Acoustic ED	6.11	6.86	6.54	8.18	6.27	16.19	6.04	6.16	5.95	7.26	5.44	6.43	138.6	282.2	222.9
Perceptual ED	0.4	0.7	0.6	0.6	0.5	0.3	0.6	0.9	0.3	0.6	0.8	0.6	0.3	0.1	0

If we compare the MZ pairs with the three most dissimilar different-speaker (DS) pairs in the acoustic analysis, taken as a control group, the difference between MZ and DS pairs in the perceptual ED is not remarkable, and this is due to the scale used here. The simplification on the perceptual protocol for voice description allows for only ten possible degrees of differentiation in a scale 0–1. Yet, the SMC of 0.3, 0.1 and 0 are very low, which agrees with the high acoustic ED. If these speaker pairs are very different between them, as shown in the acoustic analysis, scarce voice similarities are expected to be found aurally by a trained phonetician. Indeed, the values 0.3, 0.1 and 0 mean that out of 10 possible strands of voce quality variation, only three, one and none – correspondingly – sources of similarity have been found for these speakers perceptually.

Our aim has been to find how the ED in the acoustic domain correlate with ED in the perceptual domain. For that purpose, we used the Kendall’s tau correlation test, a non-parametric correlation coefficient similar to Spearman but used in preference for small data sets with certain number of tied ranks. This allowed us to assess the perceptual salience of the voice features used in the acoustic analysis.

Considering all the speakers (n = 15), results show that acoustic ED are moderately correlated with perceptual ED, *r_τ_* = −0.36, *p* < 0.05. If we only consider MZ pairs (n = 12), acoustic and perceptual ED are seldom correlated, *r_τ_* = −0.04, *p* < 0.05. As far as the interpretation of the phi-coefficient is concerned, if the agreement between the two rankings is perfect and the two rankings are the same, the coefficient has value 1; if the disagreement between the two rankings is perfect and one ranking is the reverse of the other, the coefficient has value −1. For all other arrangements the value lies between −1 and 1, and increasing values imply increasing agreement between the rankings, whereas if the rankings are independent, the coefficient has value 0.

## 4 Discussion

### 4.1 Acoustic analysis

This study investigated the potential of using pause fillers for Forensic Speaker Comparison. We demonstrated that these fillers exhibit similar acoustic characteristics to sustained vowels by examining the f0 contour and the amplitude contour. This motivated the use of speech signal processing algorithms which were originally proposed to study sustained vowels in different applications with a focus on mining information from processing signals with similar acoustic characteristics.

Therefore, we characterized each of the 875 fillers with 309 speech signal processing algorithms (voice features), and determined a robust, parsimonious subset which could jointly differentiate the two cohorts investigated, namely, MZ twins against unrelated speakers. Subsequently, we calculated acoustic ED between the speakers in our database, paired with themselves, i.e. one recording session versus another (same-speaker comparisons, SS) as well as paired with someone else (different-speaker comparisons, DS). This analysis was complemented by measuring distances between MZ pairs. Results revealed that there are significant differences among all groups. On the one hand, significant differences between DS and SS comparisons indicates overall good performance of the voice features used. It is commonly accepted in forensic phonetics [7], [8] that for a parameter to be forensically discriminant, this needs to exhibit a high degree of variation from one speaker to another (between-speaker variability) while remaining as consistent as possible for each speaker (low within-speaker variability). ASR systems are based on the same underlying idea when they compare targets and non-targets and calculate false accepts and false rejects rates, although other approaches are used to assess a forensic system [60].

On the other hand, testing the same set of features with very similar-sounding speakers, i.e. MZ pairs, provides further support for the discriminatory potential of the voice features. Our results show that similarity of MZ pairs, also measured in ED, lie between the values obtained in SS comparisons and DS comparisons. This would be due to the fact that MZ pairs are genetically identical but expected to be less similar than one individual with himself, as their anatomical plasticity – both of their vocal tract and larynx behavior – can be freely exploited by each twin member to mark differences between them. In previous studies [3] this variation leeway was found to occur more frequently in certain MZ pairs for sociolinguistic reasons than in SS comparisons. In other words, one speaker is supposed to change less from one recording session to another — with some possible exceptions, as we will discuss in relation to the biometric users called *phantoms* and *goats*.

The only MZ pair who outstood as strikingly dissimilar in the acoustic analysis (speakers 11 and 12) is the same pair who was already found less similar than the average twin pair in the likelihood-ratio investigation carried out in San Segundo [3]. A detailed diagnosis focusing on this unexpected result revealed insightful aspects in relation to both their medical anamnesis and their twin-closeness questionnaire. On the one hand, there seemed to be very different smoking habits between the twins, which together with the existence of nodules and usual sore throat in one speaker versus the other could explain their voice dissimilarities. On the other hand, the twin-closeness questionnaire revealed that they did not have an especially close relationship or were especially content with having a twin, which could have impede the most typical intratwin mimetism or accommodation, as found in the twin literature, and which would in turn have favored a voluntary tendency to vocally diverge and thus mark their own different personality. From a sociolinguistic and forensic perspective, this pair presents interesting evidence of how very similar speakers can sound very different if they intend to sound different, despite their anatomical similarities.

The same voice features were tested both under an idealized scenario of high quality recordings and also using telephone-filtered recordings, mirroring more realistic scenarios in forensic casework. Results reveal that the differences between DS, SS and MZ comparisons were significant in both high quality (HQ) and telephone-filtered (TF) recordings. Interestingly, the speakers pairs found more dissimilar under the HQ condition were not necessarily the most different pairs under the TF condition. Overall the effect exerted by the telephone filter seems to be the reduction in the differences between the speakers who were very different in HQ condition.. However, new outliers emerge in the TF condition, corresponding to speakers who were not so dissimilar in the HQ condition: 1–3, 17–19, and 34–36. In contrast, pairs 20–22 and 45–47, with high ED in the HQ condition, show ED aligned with the mean in the TF condition. This suggests that the voice features that prove useful to distinguish some speaker pairs may fail to distinguish others. It also suggests that more studies are still necessary to investigate thoroughly the effects of the telephone bandpass filtering effects telephone, which should most probably be considered in combination with the effect exerted by different codecs and compression artifacts [61].

We have also approached the question of data standardization and concluded that depending on the goal of the study and the perspective adopted, different methods for standardization could be preferred over others. While the z-score techniques may be valuable for testing comparison systems with high number of speakers without an intention to detect specific relationships between speakers, it seems that from a more traditional phonetic and sociolinguistic perspective, less reductionist techniques are better for detecting insightful detail in a similarity–dissimilarity approach while still balancing out the contribution of the most dominant variables, for instance via standardized EDs. These aspects were put in relation with the potential of heat maps to detect outlier speakers; these causing the most common misclassifications in biometric systems: false acceptances and false rejections.

The detection of possible phantoms among the speakers in a database can be used for different purposes, whether it is only to decide not to include them in the analysis because they can be thought to belong to a different population than the rest of speakers, or rather in a more front–end approach to diagnose what can cause that a speaker be so different from all others, for instance through the collection of simple questionnaires of the participants at the time of the recording, or via more detailed medical anamnesis. This would be of great importance from a traditional phonetic and sociolinguistic perspective; the interest of these disciplines lying primordially in investigating speakers’ variation patterns and finding explanations for them. For instance, previous investigations in the field of fingerprints [62] and iris recognition [63] suggest that there are few users who are intrinsically hard to match (i.e. goats and phantoms), and when they are found, their origin is either inappropriate data quality or data collection and enrollment issues, rather than any inherent characteristic of the person (cf. [57]). However, little is known about what causes the existence of phantoms and goats in speech biometrics in particular, or even if the zoo distribution is constant across different FSC systems or algorithms. Last but not least, it could be interesting to assess to which extent the speakers who outstand as very atypical in an acoustic approach are also found atypical perceptually, as we have attempted in Section 3.3.

### 4.2 Perceptual analysis

The purpose of this perceptual analysis has been twofold. On the one hand, we have explored a subfield in FSC which has not been extensively investigated in recent years. Ever since the use of acoustic software to analyze acoustic signals (e.g. f0, formant frequencies) has proliferated, these techniques have been applied to the comparison of voice samples of known and unknown origin. In contrast, auditory–perceptual methodologies remain as complementary tools to the acoustic analyses. The arrival of more sophisticated automatized methods, created ad hoc for forensic comparisons (ASR systems) and also relying on the acoustic signal, would have increased this trend. Analysis methods based on the perceptual skills of a trained expert, usually a phonetician or dialectologist, are viewed by some authors as very subjective. While it may be true that speaker discrimination which is solely based on auditory perception is error-prone, as we have explained above in relation to cognitive factors affecting the multidimensionality of a voice and as it is well acknowledged in the psychology literature, it also holds true that little has been done so far to improve on this traditional methodology. Existing protocols for the perceptual assessment of voice quality present a large leeway for – if not improvement – change toward simplification and forensic-purpose tailoring. Having noted that the VPA scheme is the most common perceptual analysis used by forensic experts nowadays [64], we have proposed a simplified version of this protocol reducing its dimensions and simplifying the original scalar degrees. This has allowed us to calculate ED between pairs of speakers in a similar way that we calculated ED in the acoustic domain.

The second objective of this preliminary perceptual analysis has been to compare the results of the acoustic analysis and the perceptual analysis, particularly with the aim of looking for correlation between both. The fact that we have found moderate correlation while only having a small number of subjects available (n = 15) suggests that the acoustic features are perceptually salient. In other words, if two speakers are found very similar based on the range of voice features derived from the acoustic signal, they are also expected to be found similar by a phonetic expert using auditory assessment of the two speakers, provided that the acoustic features have certain salience. Since our voice features depend on both vocal tract estimation and laryngeal characterization, it seems highly feasible that they are capturing the main characteristics of the individual’s voice. For instance, among the range of voice features used in this study, some cepstral coefficients would undoubtedly give an approximation of vocal tract shape. Speakers who are particularly similar based on this should be deemed similar by an expert in voice quality strands such as labial, lingual or pharyngeal settings, for instance, in our SVPA. Similarly, if features derived from the glottal source are playing the greatest role in making two speakers too similar in the acoustic domain, the phonetic expert would have rated both speakers as very similar in settings such as larynx tension or phonation types. While this idea has not been fully explored speaker by speaker in this study, the moderate correlation between acoustics and perception is good indicator that it would be possible to disentangle the role of the source features and the contribution of filter aspects in the (dis)similarity between speakers. Previous studies suggest that lack of a strong correlation between acoustic and perceptual analyses makes their combined use possible in a forensic context. For instance, French and colleagues [27] found auditory VPA (vocal tract settings only) to offer different information relevant for voice characterization than the information provided by MFCCs and LTFDs. In a forensic context this finding is important since their combination would not result in an overestimation of the strength of the evidence, as each system would be independent and, as such, encoding different types of speaker-specific information.

## 5 Conclusions and directions for future research

We have approached speaker similarity from a two-fold ‘acoustic cum auditory’ perspective. From an acoustic point of view, we have used a wide range of 309 voice signal processing features, combining source (related to the vocal folds) and filter (related to the vocal tract) voice characteristics. We have used feature selection methods to determine the most parsimonious feature subset comprising the 15 jointly most statistically predictive features, and have assessed speaker similarity on the basis of Euclidean Distances (ED). Robust assessment of similarities in voice has been undertaken for same-speaker comparisons (SS) and different-speaker (DS) comparisons; targets and non-targets in ASR ecognition terminology. The results have revealed that there are significant differences between DS and SS comparisons, which indicate good performance of the parameters for forensic identification. Besides, we have also tested the same voice features with very similar-sounding speakers, i.e. identical twins, often considered to pose a challenge for identification across different forensic disciplines; this holds also true in the voice-specific literature. The results have revealed that similarity of MZ pairs (measured in ED) lie between similarity values for SS comparisons and DS comparisons. Finally, acoustic analysis has been performed using both an idealized scenario of high quality recordings, and also telephone-filtered recordings. Results have revealed that the differences between DS and SS comparisons were significant in both high quality and telephone-filtered recordings.

From a perceptual point of view, we have proposed a preliminary simplified protocol for the perceptual assessment of voice similarity based on the VPA protocol, aimed at enabling the quantification of voice-quality features for speaker characterization and individualization purposes. The measuring of correlation between acoustic and perceptual ED have revealed that there is some agreement between acoustic and perceptual rankings, but more speakers need to be aurally assessed (preferably by more than one rater or judge) in order to obtain more robust correlation results as well as to offer measures of interrater and intrarrater agreement. This would help reduce subjectivity in this strongly human-based methodological approach and provide some indicators of reliability. All in all, the auditory assessment of voice quality still presents some challenges that need to be addressed, especially from a forensic-phonetic point of view if we want to increase not only its validity but also its reliability. Both aspects (validity and reliability) are not so well developed in this ‘acoustic cum auditory’ method as in ASR or acoustic methods, where the measurement of errors is common practice.

Although hybrid approaches to the field of FSC have been recommended by some authors for a long time (e.g. Refs. [65], [66]), not so many interdisciplinary studies can be found nowadays that approach the difficult task of speaker identification from both ASR perspectives and the more traditional linguistic approach, especially if the latter is understood as comprising auditory analyses. A notable exception is the recent investigation by González-Rodríguez et al. [67] in which two trained phoneticians undertook perceptual voice assessments of falsely accepted trials with the aim of finding how phonetic detail can be useful for the detection of differences between speakers who had been falsely identified by cepstral-only i-vector-based speaker recognition systems. Their investigation delved into the question of whether a small percentage of false acceptances in an (MFCC-based) ASR system could be avoided by using phonetic knowledge. Among other phonetic parameters, voice quality characteristics turned out to be highly relevant in speaker characterization. Potentially laryngeal voice quality features would play the greatest role, together with other features which a vocal tract-based ASR system based would not be taking into account. Our study follows a similar research line but using an established protocol for the VQ evaluation. Besides, the perceptual evaluation was not done after the acoustic analysis in order to investigate how the former could ameliorate the latter, but performed independently at two different stages.

Further directions for future work could include the perceptual assessment of speaker recordings under telephone-filter quality. Notably, false acceptances in our study mainly occur in TF condition. For instance, speaker pairs 36–38 and 46–48 are falsely identified if we fix the threshold in 5.30 which is the mean ED in SS comparisons. Perceptual evaluation of VQ in degraded conditions present a challenge that has seldom been investigated so far, but it is a forensic realistic condition worth exploring. Besides calculating inter- and intrarater agreement for the perceptual evaluation – which would require multiple raters and different rating sessions – in order to account for reliability, other future lines of research may include the weighting of VQ settings. The primary idea here is that the rarity of a setting (e.g. *tremor*, as found in Ref. [68]) should count more than a frequent category if, for example, two speakers are to be compared in a forensic context on the basis of the sum of their VQ settings. Some preliminary studies [69] are being carried out by to calculate ED between pairs of speakers based on VQ settings and taking into account this rarity-of-the-setting weighting.

From the point of view of forensic evaluation, we aim to further explore system performance using LRs. For that purpose, we will extract this same set of voice features from a larger speaker population, which will allow us to derive typicality measures from a relevant background population, and hence provide LRs as a method to evaluate the strength of the evidence.

## Footnotes

1 MATLAB source code for the computation of these algorithms is available from https://people.maths.ox.ac.uk/tsanas/.
